# Effects of Renal Denervation on Insulin Sensitivity and Inflammatory Markers in Nondiabetic Patients with Treatment-Resistant Hypertension

**DOI:** 10.1155/2017/6915310

**Published:** 2017-09-07

**Authors:** Ulla Kampmann, Ole N. Mathiassen, Kent L. Christensen, Niels H. Buus, Mette Bjerre, Henrik Vase, Niels Møller, Anne Kaltoft, Per L. Poulsen

**Affiliations:** ^1^Department of Endocrinology and Internal Medicine, Aarhus University Hospital, Nørrebrogade 44, 8000 Aarhus C, Denmark; ^2^Department of Cardiology, Aarhus University Hospital, Palle Juul-Jensens Boulevard 99, 8200 Aarhus, Denmark; ^3^Department of Nephrology, Aalborg University Hospital, Hobrovej 18-22, 9000 Aalborg, Denmark; ^4^Medical Research Laboratory, Aarhus University Hospital, Nørrebrogade 44, 8000 Aarhus C, Denmark

## Abstract

Increased sympathetic activity is important in the pathogenesis of hypertension and insulin resistance. Afferent signaling from the kidneys elevates the central sympathetic drive. We investigated the effect of catheter-based renal sympathetic denervation (RDN) on glucose metabolism, inflammatory markers, and blood pressure in nondiabetic patients with treatment-resistant hypertension. Eight subjects were included in an open-labelled study. Each patient was studied before and 6 months after RDN. Endogenous glucose production was assessed by a 3-^3^H glucose tracer, insulin sensitivity was examined by hyperinsulinemic euglycemic clamp, hormones and inflammatory markers were analyzed, and blood pressure was measured by office blood pressure readings and 24-hour ambulatory blood pressure monitoring. Insulin sensitivity (*M*-value) increased nonsignificantly from 2.68 ± 0.28 to 3.07 ± 0.41 (*p* = 0.12). A significant inverse correlation between the increase in *M*-value and BMI 6 months after RDN (*p* = 0.03) was found, suggesting beneficial effects on leaner subjects. Blood pressure decreased significantly, but there were no changes in hormones, inflammatory markers, or endogenous glucose production. Our results indicate that RDN may improve insulin sensitivity in some patients with treatment-resistant hypertension, albeit confirmation of these indications of beneficial effects on leaner subjects awaits the outcome of larger randomized controlled studies.

## 1. Introduction

Hypertension is associated with impaired glucose metabolism and insulin resistance [1], and an increased central sympathetic activity plays an important role in both conditions. Afferent signaling from the kidneys is an important contributor to the elevated central sympathetic drive, leading to hypertension, insulin resistance, heart failure, and potentially chronic kidney disease [2]. Despite the availability of many safe and effective antihypertensive drugs, blood pressure in many patients remains poorly controlled and there has been an increasing interest in the use of catheter-based renal denervation (RDN) to treat resistant hypertension [3]. The clinical results on RDN for the treatment of resistant hypertension are however diverse [3–6]. The overall effect of RDN on blood pressure is questionable, but it seems that there might be responders and nonresponders. Further studies have also been suggested to assess the effectiveness of RDN treatment for heart failure, insulin resistance, obstructive sleep apnea, atrial fibrillation, and end-stage renal disease [3]. In the current study, we investigated the effect of RDN on glucose metabolism, using gold standard methods, that is, hyperinsulinemic euglycemic clamp (HEC) and glucose isotope dilution, to assess hepatic and peripheral insulin sensitivity. As increased inflammation is associated with both hypertension and insulin resistance [7, 8], we also evaluated the effect of RDN on inflammatory markers. Accordingly, our study was designed to test whether an attenuation of the sympathetic nervous system by RDN would improve both insulin sensitivity and blood pressure and reduce inflammation.

## 2. Material and Methods

### 2.1. Study Participants

Eight patients with treatment-resistant hypertension (6 males and 2 females), defined as daytime 24-hour ambulatory blood pressure monitoring (ABPM) systolic blood pressure ≥ 145 mmHg (preceded by 1 month of scheduled drug intake showing at least 85% adherence), were included in the study. The inclusion criteria for treatment-resistant hypertension did not strictly follow AHA guidelines, as the patients in the current study were recruited from the Reset Study [9]. The study criteria are presented in Table 1. The participants were healthy, except for having treatment-resistant hypertension, as assessed by self-report, previous medical history, a physical examination, and a broad biochemical profile. Secondary forms of hypertension were excluded by means of computed axial tomography (CT) imaging of renal arteries, echocardiography, and hormone analysis. Prior to the inclusion of the current study, the 8 subjects had participated in the Reset Study [9] as part of the sham group, ensuring that drug intake was sufficient the preceding 6 months before inclusion in our study.

The participants were asked to fill in a questionnaire at baseline and at follow-up 6 months after RDN, concerning medical history, medication, smoking habits, physical activity, diet, and sleeping patterns, and they were asked not to implement changes in lifestyle during the intervention period. Changes in antihypertensive medication during follow-up were only allowed if requested by the patient or if potentially harmful changes in blood pressure arose. Study subjects were told to refrain from major physical exercise 48 hours prior to both study days.

The participants gave a written informed consent prior to the study participation. The study was conducted in accordance with the Helsinki Declaration, and the study protocol was reviewed and approved by the Regional Ethical Committee. The protocol was registered at Clinicaltrials.gov. NCT01631370.

### 2.2. Design

The study was open-labelled, where each patient was studied on two occasions, shortly before RDN and 6 months after RDN.

On the day of the study, the subjects rested on bed from 0730 h in a quiet, thermoneutral environment. The subjects fasted from the night before and during the experiments. An intravenous cannula was inserted into an antecubital vein for infusions, and another intravenous cannula was inserted into a dorsal hand vein for blood sampling. The latter was maintained heated, allowing for arterialized blood samples to be drawn. Plasma glucose levels were determined every 10 min during the clamp period, and blood samples were drawn at *t* = 0, 100, 110, 120, 220, 230, and 240 min.

At first, a 2-hour basal period (*t* = 0–120 min) was performed with infusion of a 3-^3^H glucose tracer to assess endogenous glucose production. The basal period was followed by a 2-hour clamp period (*t* = 120–240 min) with a HEC to assess insulin sensitivity. The 3-^3^H glucose tracer infusion continued during the clamp period. Indirect calorimetry was performed during the last 30 minutes of both the basal period and the clamp period.

### 2.3. Blood Pressure Measurements

Office blood pressure readings were taken in a seated position 30 minutes after the end of the study day. Averages of the triplicate measures were used. ABPM was done using either the SpaceLab 90207 or 90217 ABPM monitor with BP readings every 20 minutes. Nighttime and daytime periods were defined as 23:00–07:00 (night) and 07:00–23:00 (day). A minimum of 50% successful readings during nighttime and daytime was demanded for each ABPM to qualify for analysis.

### 2.4. Hyperinsulinemic Euglycemic Clamp

The HEC was performed to assess insulin sensitivity. Insulin (Actrapid; Novo Nordisk, Bagsværd, Denmark) was given from *t* = 120 min as a continuous infusion at 0.8 mU × kg^−1^ × min^−1^ for 120 minutes. Insulin infusates were prepared in 19 ml of isotonic saline and 1 ml of the subject's blood to prevent adsorption of insulin to plastic surfaces and infused at 5 ml/hour. Plasma glucose was clamped at 5 mmol/l by adjusting the infusion rate of 20% glucose according to plasma glucose measurements. Plasma glucose was measured every 10 min immediately after sampling. Glucose was given with a carrier infusion of 0.9% NaCl. To prevent hypokalemia, 0.22 meq/l KCl was added the glucose infusate. Steady-state plasma glucose infusion rates during the last 30 minutes of the clamp were used for estimating insulin sensitivity (*M*-value) [10, 11].

### 2.5. Tracer

For the assessment of endogenous glucose production (EGP) during both the basal and the clamp period, the 3-^3^H glucose tracer (New England Nuclear Life Science Products, Boston, MA, USA) was infused from *t* = 0–40 min (0.12 *μ*Ci/min). A priming dose of 3-^3^H glucose (12 *μ*Ci) was given as a bolus of 1.5 ml prior to the constant infusion [12]. The tracer (3–^3^ H glucose) was added to the glucose infusion during the clamp period. Glucose flux rates were calculated at 10 min intervals from *t* = 100–120 and *t* = 220–240 min [13]. Glucose rate of appearance (Ra) was calculated from Steele's equation for a nonsteady state [14, 15]. During the clamp period, EGP was calculated by subtracting the rate of exogenous glucose infusion from the rate of appearance of 3–^3^H glucose.

### 2.6. Indirect Calorimetry

The respiratory quotient (RQ) and resting energy expenditure (REE) were estimated by indirect calorimetry using a computerized flow through a canopy gas analyzer system (Deltatrac; Datex Instruments, Helsinki, Finland). Indirect calorimetry was performed during the last 30 minutes of the basal period and during the last 30 minutes of the clamp period. Mean values of the last 25 min were used for calculations. Lipid oxidation and glucose oxidation were estimated after correction for protein oxidation, which were calculated on the basis of urea nitrogen excretion [16].

### 2.7. Blood Analyses and Assays

Plasma levels of creatinine, electrolytes, lipids, HbA1c, TSH, and liver enzymes were determined by standard laboratory measures. Plasma glucose was measured in duplicate immediately after sampling on Beckman Glucoanalyzers (Beckman Instruments, Palo Alto, CA). Serum and EDTA plasma samples were frozen immediately after collection and stored at −20°C until the time of analyses.

The specific activity of 3-^3^H glucose was measured as described [17]. Insulin (DAKO, Glostrup, Denmark), C-peptide (ALPCO, Salem, NH, USA), and cortisol (EIA-1887, AH-Diagnostic) were analyzed using ELISA-based kits. Free fatty acids (FFA) were analyzed with a commercial kit (Wako Chemicals, Neuss, Germany). Plasma cytokines (interleukin- (IL-) 1*β*, IL6, IL-10, IFN-*γ*, and TNF-*α*) were analyzed by Luminex Performance Human High Sensitivity Cytokine Magnetic Panel A (Bio-Techne, Abingdon, UK). Detection limit for the analytes was between 0.3 and 1.5 pg/ml. High-sensitive C-reactive protein (hsCRP, BAM 17072, and MAB 17071, R&D Systems Europe Ltd., Abingdon, UK) was analyzed using an in-house assay. Adiponectin was analyzed by a validated in-house time-resolved immunofluorometric assay (TRIFMA) as previously described [18]. The detection limit was 1.5 *μ*g/l, and intra- and interassay coefficients of variation were <5% and <7%, respectively. Glucagon was analyzed by an in-house radioimmunoassay [19]. Total IGF-I was measured in acid ethanol-extracted serum, using an in-house TRIFMA as previously described [20].

### 2.8. Renal Denervation

RDN was carried out at one single invasive cardiovascular center and performed by one single experienced invasive cardiologist with a record of 45 procedures before initiating the current protocol. Patients were admitted in the morning and prepared for femoral artery catheterization with a 6F diagnostic catheter. Pretreatment included oral acetaminophen and 10 mg oral morphine. Unless previously examined, a coronary angiography was performed at first, in order to exclude possible asymptomatic severe proximal coronary stenosis. Thereafter, renal angiography was performed to confirm the findings from the renal CT angiography that renal artery anatomy was suitable for RDN therapy (Table 1). At this moment, sedation was administered (fentanyl, midazolam) and the Symplicity renal denervation catheter (Medtronic) was advanced, and four-to-six discrete, low-power radio frequency treatments were applied circumferentially along the length of each main renal artery, aiming at covering the entire lumen. After the procedure, patients were submitted to the ward for routine observation and were discharged in the evening or the next morning.

### 2.9. Safety

All adverse events and complication were systematically recorded. Specific interventional-related safety data included bleeding or femoral pseudoaneurysm requiring intervention, renal artery dissection, myocardial infarction, stroke, and death. Specific follow-up-related safety record concerned blood pressure, renal function, electrolyte disarrangement, stroke, transitory ischemic attack, myocardial infarction, and symptomatic hypotension.

### 2.10. Statistical Analysis

Results are expressed as mean ± SE (parametric data) or median (range) (nonparametric data). Data were tested for normal distribution using the Shapiro-Wilk test. Differences between baseline and endpoint measures and between basal and clamp data were assessed using a one-sample *t*-test or Wilcoxon signed-rank test, as appropriate. A *p* value <0.05 was considered statistically significant. Pearson correlation was used to test for correlations. Power calculations were based on the primary endpoint, namely, *M*-values, and a detectable difference in *M*-values was 14%. It could be calculated that eight patients would be required to demonstrate a significant difference at 80% power and 5% significance. All calculations were carried out using Sigma Plot version 11.0.

## 3. Results

### 3.1. Patient Characteristics and Medication

Clinical characteristics of the study subjects are presented in Table 2. Six men and 2 postmenopausal women were included. Subjects were nondiabetic with a HbA1C at 37.0 (35.0; 39.0) mmol/mol. There were no significant changes in BMI, HbA1c, fasting plasma glucose, or lipid profile before and 6 months after renal denervation. There were no changes in prescription during the six-month follow-up. Medication for cardiovascular disease and hypertension comprised the following: ace inhibitor (*n* = 2), angiotensin receptor blocker (*n* = 6), calcium channel blocker (*n* = 7), beta blocker (*n* = 3), thiazide diuretic (*n* = 3), aldosterone inhibitor (*n* = 1), alpha adrenergic blocker (*n* = 4), centrally acting sympatholytic agent (*n* = 1), acetylsalicylic acid (*n* = 5), and lipid-lowering treatment (*n* = 2).

### 3.2. Insulin Sensitivity, Endogenous Glucose Production, and Hormones

**Figure 1:**
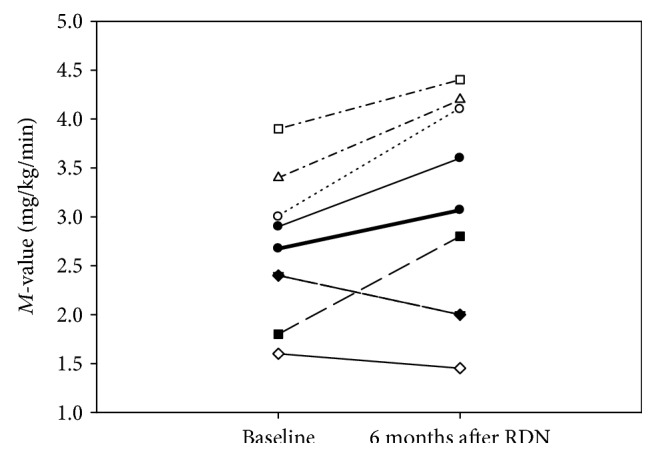
*M*-values for the 8 subjects at baseline and at 6 months after RDN. Two subjects have the same *M*-values and cannot be separated from each other in the graph (from 2.4 to 2.0 after 6 months). The thick black line depicts mean values of the *M*-values (2.86 versus 3.07; *p* = 0.12).

**Figure 2:**
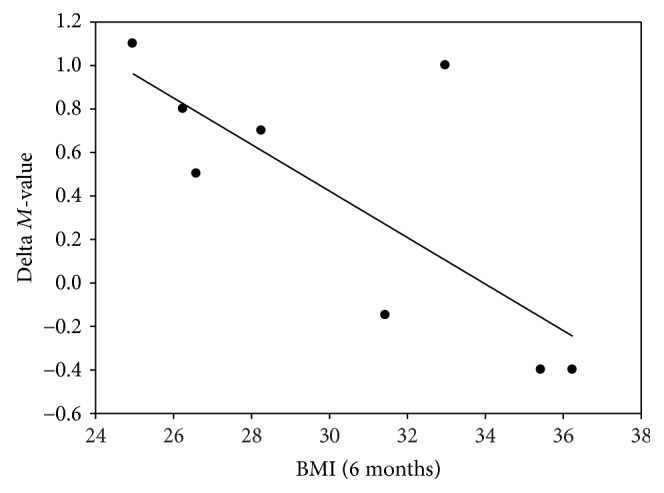
Correlation between BMI at 6 months after RDN and the difference in *M*-value before RDN and 6 months after RDN. *R* = 0.75; *p* = 0.03.

Insulin sensitivity expressed as *M*-value improved nonsignificantly from 2.68 ± 0.28 to 3.07 ± 0.41 mg/kg/min (*p* = 0.12) (Figure 1). However, a significant inverse correlation between the increase in *M*-value and BMI 6 months after RDN (*p* = 0.03) (Figure 2) was found. EGP decreased insignificantly both during the basal period (1.73 ± 0.16 versus 1.36 ± 0.19 mg/kg/min (*p* = 0.27)) and during the clamp period (0.62 ± 0.14 versus 0.36 ± 0.28 mg/kg/min (*p* = 0.52)) 6 months after RDN. There were no significant changes in C-peptide, glucagon, free fatty acids (FFA), insulin, cortisol, or IGF-I after 6 months (Table 3).

### 3.3. Systemic Inflammation

Interferon-*γ*, IL-1*β*, IL-6, IL-10, and TNF-*α* were analyzed, but as cytokine levels were very low, only IL-1*β*, IL-6, and TNF-*α* were detectable and were not affected significantly 6 months after RDN (Table 3).

### 3.4. Energy Expenditure

Fasting and insulin-stimulated resting energy expenditure (EE) and respiratory quotient (RQ) were obtained by indirect calorimetry and did not change significantly 6 months after RDN. Glucose oxidation, however, decreased significantly (*p* = 0.018) during the clamp period after RDN. Concurrently, lipid oxidation increased the borderline significantly (*p* = 0.06), whereas protein oxidation was practically unchanged (Table 3).

### 3.5. Blood Pressure Data

Six months after RDN, office systolic blood pressure and office diastolic blood pressure decreased significantly from 180.4 ± 6.79 to 164 ± 8.06 (*p* = 0.01) and 94.88 ± 4.72 to 87.88 ± 3.63 (*p* = 0.04), respectively. There were a significant borderline decrease in nighttime ABPM systolic blood pressure (*p* = 0.06) and a significant decrease in nighttime ABPM diastolic blood pressure (*p* = 0.03) (Table 4).

## 4. Discussion

This study, based on a small sample size, showed no overall effect of RDN on insulin sensitivity, assessed by the HEC. The significant inverse correlation between the increase in *M*-value and BMI 6 months after RDN could indicate that RDN could have a potential effect on nonobese subjects, without severe insulin resistance. This is in a way a puzzling finding as obesity contributes to an increase in sympathetic nervous system activity and obesity itself contributes to insulin resistance. However, one could speculate that the most obese subjects in the current study are so insulin resistant due to their obesity that the renal denervation is relatively less effective compared to the leaner subjects that are less insulin resistant. Our findings are in line with the recently published study by Miroslawka et al. [21] where 23 patients with treatment-resistant hypertension underwent RDN and a two-step HEC with glucose tracer infusion was performed before and 6 months after RDN. The authors found no improvement in insulin sensitivity, but 18 subjects had the metabolic syndrome and the mean BMI for all 23 patients was 32 kg/m^2^, compared to only 29 kg/m^2^ in our study. In addition, Miroslawka et al. found an insignificant decrease in EGP, whereas we found an insignificant decrease in EGP both during the basal and the clamp period 6 months after RDN, compatible with a modest improvement in hepatic insulin sensitivity. This is supported by a recent study, in which it was found that in a nonhypertensive obese canine model, RDN completely normalized hepatic insulin sensitivity (EGP), assessed by the HEC, in high-fat diet-fed animals, compared to sham animals. The authors accordingly suggested that the renal nerves play a role in the regulation of insulin action specifically as regards EGP [22]. In our study, glucose oxidation also decreased significantly during the clamp period after RDN whereas lipid oxidation concurrently increased the borderline significantly, possibly as a result of a lower EGP after RDN.

In parallel with the Symplicity HTN-2 trial [4], we found a significant decrease in office-based blood pressure measurements after RDN. However, the decrease in blood pressure could relate to inadequacies of the study design, as subsequent more rigorously designed clinical trials using a sham procedure and ABPM have failed to demonstrate a BP-lowering effect [5, 6, 9]. In the current study, it is plausible that systolic office blood pressure falls significantly as the subjects are more used to the procedures on the second day of examination. Therefore, the 24-hour ambulatory blood pressure measurements are much more valid.

Hypertension is associated with the infiltration of T cells into the kidney and vasculature, with the release of cytokines, including IFN-*γ* and TNF-*α*, which promote sodium retention, vasoconstriction, and oxidative injury [7]. Obesity causes lipid accumulation in adipocytes that can increase the production of proinflammatory cytokines such as TNF-*α*, IL-6, and IL-1*β*, and this obesity-associated chronic low-grade inflammation leads to insulin resistance [23]. Moreover, increased levels of inflammatory markers such as CRP and white cell blood count correlate with incident type 2 diabetes [8]. In our study, we could not demonstrate an improvement in inflammatory markers 6 months after RDN; it should however be noted that the levels of some cytokines before RDN were below detection limit, making detection of any change analytically problematic.

One of the limitations of the study is the small sample size, although 8 subjects should suffice for fulfilling the purpose of the study. Power calculations were based on the primary endpoint, namely, the *M*-values found in the hyperinsulinemic euglycemic clamp. Clamp studies are labour intensive and technically demanding and are therefore most commonly conducted in a restricted number of subjects, often including around 8–10 subjects. Moreover, the method had only been applied to 2 subjects treated with RDN [24] when our study was designed, and therefore, there was no precedent that allowed us to compare our results or power calculations with those of other studies. Later, Miroslawka et al. [21] published a study including 23 subjects with treatment-resistant hypertension who were examined by an HEC before and 6 months after RDN. However, as mentioned previously, most of the participants in the study suffered from the metabolic syndrome and were more obese than the participants in our study.

Another limitation is that we used the unipolar Symplicity Flex catheter and not a multipolar catheter in our study. Accordingly, we cannot exclude that the failure of RDN to improve insulin resistance in all 8 subjects is due to insufficient ablation or recurrence due to nerve regeneration after 6 months.

Using an open-labelled study design, the significant reduction in blood pressure and the partial improvement in insulin sensitivity could be explained by the positive “placebo” effects of being included in a study, potentially leading to a greater awareness of a healthier lifestyle and a better compliance to medication. The observed reductions in blood pressure should therefore be interpreted cautiously as previously mentioned.

Another point of criticism could also be that both males and females were included in the study. However, as we only included postmenopausal women, we considered them to be comparable to the men in our study group. Moreover, we performed a subanalysis only including the men, and the data on the most important outcomes (*M*-value and blood pressure) only showed a significant change in office diastolic blood pressure, but as 24-hour ambulatory blood pressure measurements are more precise, we do not find the change highly relevant.

On the other hand, our study design also has strengths. One single, highly skilled, and experienced invasive cardiologist performed the RDN, potentially increasing the rate of success. In addition, to assess insulin sensitivity, we used the gold standard method, HEC, and to assess hepatic insulin sensitivity, we used a glucose tracer. Until recently, all studies focusing on the effects of RDN on glucose metabolism have used less suited measures like HOMA to assess insulin sensitivity. Thus, in the study by Mahfoud et al., 50 patients with treatment-resistant hypertension were included in a study where 37 patients underwent RDN and 13 patients comprised a control group. Forty percent of the participants had type 2 diabetes before inclusion in the study. Fasting glucose, insulin, C-peptide levels, and insulin resistance assessed by HOMA-IR decreased significantly 3 months after RDN, whereas there were no significant changes in the control group [25]. In the DREAMS study by Verloop et al., 29 patients with the metabolic syndrome were treated with RDN. Insulin sensitivity was assessed by the simple index assessing insulin sensitivity through the oral glucose tolerance test and did not change 6 and 12 months after RDN [26].

Also, in the studies just mentioned, most of the participants had diabetes or prediabetes, making the study groups more heterogeneous compared to subjects in our study.

To our knowledge, no other study has assessed the effects of RDN on inflammatory markers, a parameter that could be a contributor to the association between hypertension and insulin resistance. In addition, the finding that RDN may improve insulin sensitivity in leaner subjects is also of novelty.

## 5. Conclusions

In this relatively small, nonrandomized study, using gold standard methods to assess insulin sensitivity, there were no significant changes in basal and insulin-stimulated glucose disposal and EGP after RDN. We did however find an overall trend towards improved insulin sensitivity largely driven by more pronounced changes in leaner subjects, suggesting that RDN may be specifically beneficial in this subgroup.

Ideally, reducing sympathetic tone by RDN could improve both blood pressure and insulin sensitivity, thus targeting two important risk factors of cardiovascular disease. Currently, the challenge lies in identifying subgroups that could benefit from RDN. For now, there is not enough evidence to expand the indication for RDN to include insulin resistance, as further and larger studies are needed.

## 6. Limitations

The most important limitation of the current study is the small sample size, but as previously mentioned, there was no precedent that allowed us to compare our results or power calculations with those of other studies when our study was designed. Power calculations were based on the primary endpoint, the *M*-values found in the hyperinsulinemic euglycemic clamp. The SD of the *M*-value has been shown to be 10% in previous studies in our laboratory, and *λ* is the difference that we wished to detect, in this case 14%. The power calculation was therefore *N* = 2 × 7.9 × (10/14)^2^ = 8. Consequently, eight patients would be required to demonstrate a significant difference at 80% power and 5% significance.

Another important limitation of the study is the fact that we included both men and postmenopausal women in our study, but as described in the study by Wassertheil-Smoller et al. [27], women aged <55 years tend to have lower prevalence rates of hypertension, compared with men, but women aged 55 to 74 years have similar rates. The women in our study were aged between 55 and 74, making them comparable to the men in our study group. We, however, performed subanalyses only including the men, but the results did overall not change significantly. We therefore decided to pool male and female patients in order to get a larger “*n*.”

## Figures and Tables

**Table 1:** Study criteria.

Inclusion
(i) Age 30 to 70 years
(ii) One month of stable antihypertensive treatment with at least three antihypertensive agents including a diuretic (or in case of diuretic intolerance, a minimum of three nondiuretic antihypertensive drugs)
(iii) Daytime ABPM systolic blood pressure ≥ 145 mmHg (preceded by 1 month of scheduled drug intake showing at least 85% adherence)
Exclusion
*General*
(i) Noncompliant personality (abuse, mental illness)
(ii) Pregnancy/inadequate contraception in fertile women
(iii) Known allergy to iodine-containing X-ray contrast agent
*Comorbidity*
(i) Diabetes
(ii) Secondary hypertension
(iii) Malignant disease
(iv) Congestive heart failure NYHA 3-4
(v) Chronic renal failure stages 4-5 (eGFR ≤ 30 ml/min/1.73m^2^)
(vi) Stable angina pectoris (CCS classes 2–4)
(vii) Unstable angina pectoris
(viii) Coronary artery disease with indication for coronary intervention
(ix) Recent myocardial infarction or coronary intervention (<6 months)
(x) Permanent atrial fibrillation
(xi) Orthostatic syncope (<6 months)
(xii) Symptomatic peripheral artery disease
*Paraclinical*
(i) Clinically significant abnormal electrolytes and liver function tests.
(ii) Hemoglobin < 7.0 mmol/l
(iii) Abnormal thyroid function
(iv) Macroscopic haematuria
(v) ECG: AV-block grades 2 and 3 or AV-block grades 1 + branch block
*Echocardiography*
(i) Left ventricular ejection fraction < 50%
(ii) Significant valvular disease
*CT-angiography and selective angiography of renal arteries*
(i) Pronounced calcification in iliaco-aortic or renal arteries
(ii) Multiple renal arteries: accessory renal arteries estimated to carry more than 10% of the kidney's blood supply (small polar arteries accepted) and being undersized (see below) for ablation procedure
(iii) Renal artery diameter < 4 mm
(iv) Renal artery length (from ostium to first major side branch) < 20 mm
(v) Renal artery disease (stenosis, fibromuscular dysplasia; prior intervention, dissection)

**Table 2:** Anthropometric and biochemical measures.

Characteristics	Baseline	6 months after RDN	*p* value
Men/women	6/2		
Age (years)	62.5 ± 2.55		
BMI (kg/m^2^)	28.9 (26.6; 34.0)	29.9 (26.4; 34.2)	0.94
Fasting plasma glucose (mmol/l)	6.31 ± 0.35	6.14 ± 0.31	0.29
HbA1c (mmol/mol)	37.0 (35.0; 39.0)	37.5 (36.0; 40.5)	0.58
P-sodium (mmol/l)	142.4 ± 0.68	141.9 ± 0.74	0.52
P-potassium (mmol/l)	3.45 (3.15; 3.60)	3.55 (3.40; 3.65)	0.06
P-total cholesterol (mmol/l)	5.26 ± 0.41	5.25 ± 0.36	0.91
P-LDL cholesterol (mmol/l)	3.18 ± 0.34	3.19 ± 0.31	0.94
P-HDL cholesterol (mmol/l)	1.33 ± 0.11	1.34 ± 0.14	0.74
P-triglycerides (mmol/l)	1.45 (1.05; 2.25)	1.35 (1.10; 1.95)	0.94
P-TSH (×10^−3^ ie/l)	1.95 (1.36; 2.81)	1.80 (1.40; 2.73)	0.38
P-ALAT (I/U)	23.50 ± 3.82	23.63 ± 4.83	0.93
P-creatinine (*μ*mol/l)	86.88 ± 7.52	84.38 ± 7.62	0.53
eGFR (ml/min)	72.38 ± 4.80	76.00 ± 4.67	0.31
B-leucocytes (×10^9^/l)	5.36 ± 0.39	5.49 ± 0.46	0.76
B-thrombocytes (×10^9^/l)	194.4 ± 15.9	188.1 ± 14.6	0.40
B-hemoglobin (mmol/l)	9.04 ± 0.26	8.93 ± 0.24	0.41

All data are presented as means with SE or median (range).

**Table 3:** Circulating hormones, cytokines, and metabolic parameters.

	Baseline values		6 months after RDN		*p* value	*p* value	*p* value	*p* value
	Basal^∗^	Clamp^∗∗^	Basal	Clamp	Difference between basal and clamp values at baseline	Difference between basal and clamp values 6 months after RDN	Difference between basal values at baseline and 6 months after RDN	Difference between clamp values at baseline and 6 months after RDN
M-value (mg/kg/min)		2.68 ± 0.28		3.07 ± 0.41				0.12
P-C-peptide (pmol/l)	795.0 ± 121.8	466.0 ± 89.86	920.4 ± 209.7	529.3 ± 124.7	0.001	0.009	0.32	0.18
P-glucagon (pg/ml)	67.13 ± 5.81	48.63 ± 4.35	65.50 ± 6.99	45.88 ± 4.36	<0.001	<0.001	0.73	0.47
P-FFA (mmol/l)	0.52 ± 0.05	0.06 ± 0.009	0.53 ± 0.07	0.05 ± 0.012	<0.001	<0.001	0.88	0.14
P-insulin (pmol/l)	81.88 ± 13.41	500.0 ± 63.53	92.50 ± 11.56	495.8 ± 75.51	<0.001	0.008	0.20	0.88
P-cortisol (ng/ml)	137.3 ± 15.72		145.6 ± 15.53				0.46	
IGF-I (ng/ml)	124.3 ± 9.79		132.8 ± 12.21				0.14	
Adiponectin (mg/l)	12.94 ± 0.79		12.72 ± 1.16				0.64	
hsCRP (mg/l)	4.60 ± 1.28		4.99 ± 1.14				0.65	
TNF-*α* (pg/ml)	4.62 ± 0.93		5.21 ± 0.81				0.21	
IL-6 (pg/ml)	0.64 ± 0.11		0.67 ± 0.09				0.78	
IL-1*β* (pg/ml)	0.04 ± 0.006		0.05 ± 0.01				0.15	
EGP (mg/kg/min)	1.73 ± 0.16	0.62 ± 0.14	1.36 ± 0.19	0.36 ± 0.28	0.002	0.004	0.27	0.52
EE (kcal)	1950 ± 108	1917 ± 114	1901 ± 105	1881 ± 96	0.19	0.48	0.43	0.53
RQ	0.82 ± 0.01	0.88 ± 0.01	0.84 ± 0.01	0.88 ± 0.02	0.007	0.03	0.43	1.00
Glucose oxidation. (mg/kg/min)	1.11 ± 0.21	1.58 ± 0.13	1.31 ± 0.28	0.92 ± 0.21	0.06	0.22	0.62	0.018
Protein oxidation (mg/kg/min)	0.69 ± 0.05	0.67 ± 0.05	0.74 (0.65;1.09)	0.86 (0.59;0.94)	0.98	1.0	0.26	0.36
Lipid oxidation (mg/kg/min)	0.65 ± 0.06	0.42 ± 0.08	0.55 ± 0.05	0.78 ± 0.12	0.002	0.19	0.29	0.06

^∗^Basal is at time = 0 min except for EGP (endogenous glucose production), where basal is at time = 120 min; ^∗∗^clamp is at time = 240 min. All data are presented as means with SE or median (range).

**Table 4:** Blood pressure measurements.

	Baseline	6 months after RDN	*p* value
Office systolic blood pressure (mmHg)	180.4 ± 6.79	164.8 ± 8.06	0.01
Office diastolic blood pressure (mmHg)	94.88 ± 4.72	87.88 ± 3.63	0.04
Daytime ABPM systolic (mmHg)	158.3 ± 4.72	154.4 ± 5.53	0.49
Daytime ABPM diastolic (mmHg)	89.43 ± 2.84	88.29 ± 4.32	0.74
Nighttime ABPM systolic (mmHg)	151.3 ± 5.78	138.1 ± 8.00	0.06
Nighttime ABPM diastolic (mmHg)	83.71 ± 2.80	75.57 ± 4.64	0.03

All data are presented as means with SE or median (range). ABMP: 24-hour blood pressure measurements (*n* = 7).

## Abbreviations

RDN: Renal denervation
HEC: Hyperinsulinemic euglycemic clamp
ABPM: 24-hour ambulatory blood pressure monitoring
EGP: Endogenous glucose production
CT: Computed axial tomography
Ra: Rate of appearance
RQ: Respiratory quotient
REE: Resting energy expenditure
FFA: Free fatty acids.
